# Peptide Inhibitor Assay for Allocating Functionally Important Accessible Sites Throughout a Protein Chain: Restriction Endonuclease EcoRI as a Model Protein System

**DOI:** 10.3390/biotech14010001

**Published:** 2024-12-30

**Authors:** Joji M. Otaki

**Affiliations:** The BCPH Unit of Molecular Physiology, Department of Chemistry, Biology and Marine Science, Faculty of Science, University of the Ryukyus, Nishihara 903-0213, Okinawa, Japan; otaki@sci.u-ryukyu.ac.jp; Tel.: +81-98-895-8557

**Keywords:** molecular accessibility, EcoRI, peptide inhibitor, protein structure and function, drug design, peptide and protein engineering

## Abstract

Functionally important amino acid sequences in proteins are often located at multiple sites. Three-dimensional structural analysis and site-directed mutagenesis may be performed to allocate functional sites for understanding structure‒function relationships and for developing novel inhibitory drugs. However, such methods are too demanding to comprehensively cover potential functional sites throughout a protein chain. Here, a peptide inhibitor assay (PIA) was devised to allocate functionally important accessible sites in proteins. This simple method presumes that protein‒ligand interactions, intramolecular interactions, and dimerization interactions can be partially inhibited by high concentrations of competitive “endogenous” peptides of the protein of interest. Focusing on the restriction endonuclease EcoRI as a model protein system, many endogenous peptides (6mer-14mer) were synthesized, covering the entire EcoRI protein chain. Some of them were highly inhibitory, but interestingly, the nine most effective peptides were located outside the active sites, with the exception of one. Relatively long peptides with aromatic residues (F, H, W, and Y) corresponding to secondary structures were generally effective. Because synthetic peptides are flexible enough to change length and amino acid residues, this method may be useful for quickly and comprehensively understanding structure‒function relationships and developing novel drugs or epitopes for neutralizing antibodies.

## 1. Introduction

Functional three-dimensional protein structures are maintained dynamically via intramolecular interactions and protein‒protein interactions [1,2]. Such structure‒function relationships in proteins are important for understanding the molecular mechanisms of protein activities and rationally developing protein inhibitors for biochemical research and medicine. Protein functional sites may include substrate recognition sites, catalytic active sites, allosteric sites for conformational change, protein‒protein interaction sites, and other important sites to achieve specific structures and functions. Among the various approaches to understanding protein molecules and discovering novel drugs, peptides and their derivatives have been proposed to be useful, and this approach is known as peptidomimetics [3,4,5,6,7,8,9]. For example, combinatorial synthetic peptide arrays and libraries may be used to capture low-abundance proteins, understand protein‒ligand interactions, and develop novel peptidomimetic drugs [10,11]. These peptide-based techniques may be useful, especially for peptide receptors, including some G-protein-coupled receptors [12], but a similar idea can be applied not only to peptide receptors but also to any protein in general.

Among various intramolecular and intermolecular interactions, protein‒protein interactions (PPIs) are considered excellent targets of peptide ligands for drug development [12,13,14,15,16,17]. For example, α-helix-mediated PPIs may be targeted for potential inhibitory sites in various proteins [18]. In contrast, a β-sheet region of the HIV protease has been targeted to inhibit the dimerization of this protein by peptide ligands [19]. PPI inhibitors may be screened through phage-displayed peptide libraries [20,21]. These peptide ligands may also be designed rationally in silico in reference to three-dimensional structures of potential binding sites [22,23,24,25,26,27,28]. For example, recent developments in CIP (cancer immune checkpoint) peptide inhibitors have been performed computationally and screened through random peptide libraries [7]. PPI inhibitory peptides may be designed from the interacting short constituent sequences (SCSs) of target proteins, but the isolated “endogenous” peptides do not usually fold into secondary structures. To make endogenous peptide inhibitors more efficient, peptide conformation should be constrained by incorporating functional groups or artificial backbone into peptides [29].

There are many three-dimensional structural data of proteins available in the Protein Data Bank (PDB) [30,31], and in many cases, major functional sites of proteins have been structurally visualized. These data provide the foundations for understanding protein functions and rational drug design. Furthermore, even if three-dimensional structural data are not available in the PDB, the primary amino acid sequences in proteins may be used to predict three-dimensional structures and functional sites with high precision in silico via an AI-based machine called AlphaFold [32], then AlphaFold 2 [33] and AlphaFold 3 [34], on the basis of which potential inhibitors may be designed rationally. However, experimental validation of functional sites in vitro is still important. To do so, site-directed mutagenesis [35,36] may be the method for validating functional sites at the amino acid level. Saturation mutagenesis at several candidate sites may often be needed. However, such mutagenesis experiments are demanding because there are almost infinite possibilities if all sequences are to be covered combinatorially, even though structural predictions of proteins certainly help researchers narrow down candidate sites. A more fundamental disadvantage of conventional methods is that even if a functional site is discovered and examined in detail via these conventional methods, there may be other functional sites yet to be discovered in a known protein. Moreover, protein functional sites may be intrinsically disordered [37,38,39,40,41]. In that case, the conventional methods above certainly miss intrinsically disordered functional sites because no rigorous three-dimensional structures are assigned to intrinsically disordered regions.

**Figure 1:**
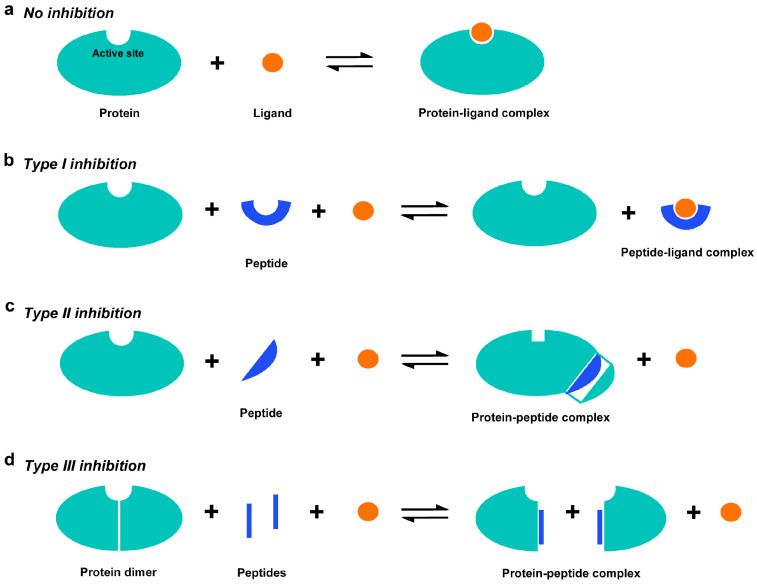
Three possible types of peptide inhibition via synthetic endogenous peptides in the peptide inhibitor assay (PIA). (**a**) No inhibition. A ligand binds to the active site (functional site) of a protein, forming a protein‒ligand complex. (**b**) Type I inhibition: competitive binding of a peptide to a ligand (substrate) because a peptide mimics the active site of the protein. (**c**) Type II inhibition: competitive binding of a peptide to a site of intramolecular interactions. (**d**) Type III inhibition: competitive binding of a peptide to a dimerization site or a site of protein‒protein interaction (PPI).

This study presents a peptide inhibitor assay (PIA) for allocating functionally important accessible sites throughout a protein molecule. PIA is a simple, fast, and exhaustive method for investigating functional inhibition by synthetic endogenous peptides (called blocking peptides or BPs in this study) that are derived from short constituent sequences (SCSs) of a target protein itself. The idea of PIA has been derived theoretically as described below. A protein generally forms a protein‒ligand complex to execute its function (Figure 1a). When a peptide has an amino acid sequence identical to a functional site of a target protein, this peptide may partially mimic the functional site and competitively bind to ligand molecules, consequently lowering the enzymatic efficiency (Figure 1b). This type of peptide inhibition may be called type I. Because a functional site is often composed of several amino acid residues from different linear positions, type I inhibition by a single peptide is expected to be rare. When a peptide has an amino acid sequence important for three-dimensional structure and functional integrity, intramolecular interactions between amino acids may be competitively inhibited by the binding of the peptide to the corresponding amino acid sequence of the target protein, consequently lowering the functional integrity of the protein (Figure 1c). This type of peptide inhibition may be called type II. In that case, the competitive sites may not necessarily be catalytically active sites, but nonetheless are important for protein folding and functions. Type II inhibition may be found in peptides corresponding to β-sheet structures due to hydrogen bonds connecting two adjacent strands, but peptides corresponding to α-helices interacting with other parts of the same protein may also function as type II inhibitors. When a peptide has an amino acid sequence identical to the dimerization interface, this peptide may competitively bind to that site, consequently inhibiting dimerization and catalytic functions if dimerization is critical for catalytic activity (Figure 1d). This type of peptide inhibition may be called type III, and it is this type III inhibition that is an active research field for developing PPI inhibitors, as mentioned above. In any case, if a synthetic peptide shows an inhibitory effect, then the amino acid sequence used for that peptide is considered a candidate functional site or structurally important site in the protein. The type II and type III peptide inhibitors do not compete for active sites in most cases, but they may also be considered “competitive” inhibitors in a broad sense.

Because peptides do not stably fold to mimic endogenous secondary structures, the inhibitory effects of endogenous peptides are likely limited and difficult to detect under the optimum conditions for proteins of interest. Thus, to detect an inhibitory effect, PIA may be performed under conditions that are not optimal for the proteins of interest. Nonetheless, when a high concentration of an endogenous peptide is used, a portion of the endogenous peptide molecules may probabilistically be able to form endogenous secondary structures for a very short time even if that structure may be thermodynamically unfavorable in the steady state. As long as these considerations are met, PIA may be practically attainable. The validity and significance of the inhibitory effects under nonoptimum conditions may be arguable, but the PIA results under nonoptimum conditions should be considered a first step in identifying functional sites, and thus in the development of novel drugs. Another support for PIA comes from a group of studies that stresses the important contribution of short constituent sequences (SCSs) of proteins to secondary structures and the functionality of proteins [42,43,44,45,46,47,48,49,50,51,52,53,54]. A well-known structural prediction approach, ROSETTA, is also based on data collection of SCSs from the PDB [55,56].

Importantly, PIA does not require any three-dimensional information to investigate candidate functional sites. Equally importantly, PIA can comprehensively cover the overall amino acid sequences of a protein throughout a protein chain, which may be difficult, if not impossible, in mutagenesis-based methods. PIA may be improved to be a high-throughput system once an enzymatic assay system is established. This method can identify only sites accessible for peptides, and nonaccessible sites may not be discovered via this method. For the purpose of screening candidate drugs and epitopes for neutralizing antibodies, this accessibility bias may be convenient for researchers because only accessible sites are valid for functional inhibition by drugs. In any case, PIA may be a starting method for quickly searching for candidate functional sites in proteins.

In this study, we focused on the well-known restriction endonuclease EcoRI as a model protein system [57]. EcoRI is a type II restriction endonuclease that recognizes the short palindromic DNA sequence GAATTC and cleaves the recognition site in the presence of Mg^2+^ ions as homodimers [58]. It is one of the most common restriction enzymes used for the molecular cloning of DNA fragments. EcoRI, like other type II restriction endonucleases, binds to DNA nonspecifically and scans DNA for specific binding sequences through linear diffusion. This linear diffusion with nonspecific binding to DNA is faster than diffusion proper, and is thus called facilitated diffusion [59]. Facilitated diffusion seems to have two parts, sliding and hopping, and the latter requires high ionic strength [60], which may be assisted by a quantum walk mechanism [61]. After facilitated diffusion, specific binding to GAATTC induces a conformational change in EcoRI and DNA, resulting in the activation of the catalytic reaction [62].

**Figure 2:**
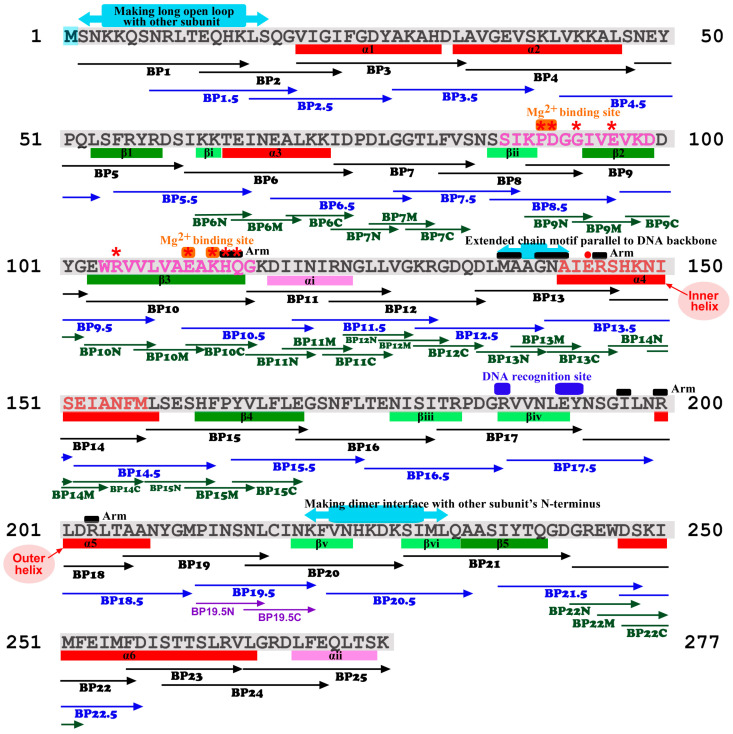
Amino acid sequence of EcoRI (M1-K277). The first M is removed after translation in this protein. The secondary structures and functional sites are indicated below and above the EcoRI sequence, respectively, according to Kim et al. (1990) [63], Heitman (1992) [64], and Watrob et al. (2001) [78]. The blocking peptides (BPs) designed and tested in the present study are indicated below the EcoRI sequence. Major active site sequences are indicated in pink letters (SIKPDGGIVEVKD and WRVVLVAEAKHQG). Red asterisks indicate important sites identified via site-directed mutagenesis. A red dot (E144) indicates salt bridge formation. A sequence of EcoRI corresponding to a helical peptide (α4) that has been demonstrated for its inhibitory effect on EcoRI by Brickner and Chmielewski (1998) [81] is indicated in red letters (AIERSHKNISEIANFM). Two parallel helices, α4 and α5, that form the inner and outer helices, respectively, to hold DNA are indicated.

To allocate functional sites, as is the case for other proteins, the crystal structures of EcoRI [62,63] have been the basis for site-directed mutagenesis [64]. Amino acid sequence of EcoRI with secondary structures and known functional sites are shown in Figure 2. Crystal structures indicate that R200 binds to guanine and that E144 and R145 form hydrogen bonds with the adenine of the cognate DNA sequence [64]. Indeed, site-directed mutagenesis confirmed that catalytic activity, but not specificity, decreased in E144Q and R145K [65]. Similarly, the specificity of mutants with R200K, E144Q + R145K, and E144Q + R145K + R200K did not change, but the catalytic activity decreased [66,67,68]. Furthermore, D59 is not important for catalysis [69]. M137 functions for specific DNA recognition but not I197 [70]. Site-directed mutagenesis at Q115 [71] and at P90, D91, E111, and K113 at the catalytic center has been shown to be essential for catalysis [72]. Temperature-sensitive mutants generated via random mutagenesis have been analyzed: G78D and A235E affected the protein–protein interaction (PPI) for dimerization, and P90S affected the active site [73]. The promiscuous mutant A138T binds more tightly to the cognate DNA sequence than does wild-type EcoRI but has higher star activity at AAATTC [74,75]. H114Y and its saturation mutagenesis revealed that H114 contributes to both recognition and catalysis [76]. Additionally, N141 has been shown to be essential for specific cognate DNA recognition [77]. On the other hand, the N-terminus of EcoRI has not been visualized in crystal structures. Solution structures revealed conformational changes in EcoRI when binding to DNA and Mg^2+^, and that the N-terminus plays an important role in dimerization [78,79].

While the structure‒function data on EcoRI have accumulated over decades, as mentioned above, there seem to be no systematic studies that have scanned the entire EcoRI protein chain for possible functional importance. Importantly, dimeric PPIs and specific binding to the cognate DNA sequence of EcoRI can be inhibited by peptides designed from an α-helix region called α4 [80,81]. To characterize EcoRI further, PIA was performed to cover the entire protein chain of EcoRI in the present study. The objective of the present PIA is to examine all possible functional sites with high accessibility, although roughly, throughout an EcoRI molecule via multiple endogenous peptides. The present PIA results were compared with the currently available knowledge of the functional allocation of EcoRI.

## 2. Materials and Methods

### 2.1. Plasmid

A recombinant plasmid, pCRII-TOPO (Invitrogen, Waltham, MA, USA), containing a PCR fragment of the full-length cDNA (978 bp) of the mouse odorant receptor OR23 [82,83,84,85,86] at the EcoRI cloning site (GAATTC) was used as a DNA substrate for EcoRI. OR23 does not contain any cognate DNA sequence of EcoRI. This pCRII-TOPO-OR23 plasmid was prepared from TOP10F’ *Escherichia coli* (Invitrogen) using ATP Plasmid Mini Kit (ATP Biotech, Taipei, ROC). The plasmid solution was prepared at 40 ng/μL by adding autoclaved DNase-free RNase-free deionized H_2_O (Nippon Gene, Tokyo, Japan). The DNA concentrations were measured with a Qubit fluorometer (Invitrogen).

### 2.2. EcoRI Sequence and Synthetic Peptides

The amino acid sequence of EcoRI was obtained from the PDB (PDB ID: 1ERI) [63]. The peptides were designed to cover all regions of the amino acid sequence of EcoRI. The original 25 blocking peptides (BP) were designed to roughly correspond to secondary structures and to cover the entire EcoRI protein chain with these 25 BPs (Table 1). Randomized peptides were designed on the basis of the sequences of BP7, BP10, and BP13 (Table 2). BP7, BP10, and BP13 were subjected to the randomization analysis because they had medium-level inhibitory effects, which may be convenient to detect changes in inhibitory effects after randomization. The randomization process was manually executed by drawing amino acid cards. A total of 22 additional staggered peptides were designed to have N-terminal and C-terminal regions of the two consecutive original BPs (Table 3). Regarding an unexpected effect of BP19.5, its deletion peptides (BP19.5N and BP19.5C) were designed and synthesized (Table 4). Furthermore, 30 short BPs were designed to have N-terminal (N), middle (M), and C-terminal (C) regions of the original BPs (Table 5). In total, 85 peptides (79 “endogenous” peptides and 6 randomized peptides) were designed and synthesized (Figure 2).

Peptide synthesis was performed by GenScript Japan (Tokyo, Japan). All peptides were confirmed to be more than 75% pure via high-performance liquid chromatography (HPLC) and to have theoretical molecular weights via mass spectrometry (MS). The peptides were dissolved in DMSO (dimethyl sulfoxide) and were prepared at 5.59 × 10^−9^ mol/μL (5.59 mM). When 1.0 μL of this peptide mixture was mixed in a tube for the inhibition of EcoRI catalytic activity, the molar ratio of EcoRI to peptide was 1:500. This concentration in molar equivalence was used throughout this study unless otherwise noted.

Several physicochemical factors for synthetic peptides were obtained. Molecular weight, theoretical isoelectric pI, mean hydrophobicity (grand average of hydropathicity (GRAVY) (Table 1, Table 2, Table 3, Table 4 and Table 5), aliphalic index, and instability index (II) (Table 6) were obtained from Expasy’s ProtParam [87] at https://web.expasy.org/protparam (accessed on 20 November 2024). Net charge at pH7 and the percentage of the number of hydrophilic residues in the total number of residues (Table 6) were obtained from Peptide Calculator of Bachem AG (Bubendorf, Switzerland) at https://www.bachem.com/knowledge-center/peptide-calculator/ (accessed on 20 November 2024). The number of aromatic side chains (F, H, W, and Y) (Table 6) was manually counted.

### 2.3. Enzymatic Digestion with EcoRI

Purified and concentrated EcoRI (20,000 U/mL, approximately 338 μg/mL, 1.12 × 10^−2^ nM) was obtained from New England Biolabs (Ipswich, MA, USA). To quantify and visualize the inhibitory effects of the peptides, the enzymatic reaction conditions were set so that at least three hours of incubation were required to completely digest the substrate plasmid DNA with EcoRI. To do so, we used a low Mg^2+^ concentration (0.015 mM) in the reaction mixture in contrast to the optimum concentration of Mg^2+^ for EcoRI (10 mM).

All master solutions and samples were prepared on ice. A single reaction tube contained the following common ingredients: autoclaved DNase-free RNase-free deionized H_2_O (12.5 μL) (Nippon Gene), 10× NE Buffer Mg^2+^-free (2.0 μL), pCRII-TOPO-OR23 plasmid DNA (2.0 μL of 40 ng/μL solution), MgCl_2_ (1.5 μL of 0.20 mM solution), and EcoRI (1.0 μL). A master solution containing these ingredients without DMSO or a peptide was first prepared for the number of sample tubes. The master solution was aliquoted into 1.5 mL spin tubes (19.0 μL per tube). Then, 1.0 μL of DMSO or a peptide sample was added, and the mixture was incubated at 37 °C. A negative control with DMSO but without a peptide (“no peptide” control) was run in every assay. When the digestion efficiency was compared, another negative control with autoclaved DNase-free RNase-free H_2_O without EcoRI was prepared. After the reaction, the samples were quickly cooled on ice for ten minutes and then stored at −80 °C until agarose gel electrophoresis. The above protocol was conducted for all peptides (including BP19.5, BP19.5N, and BP19.5C) used in the present study unless otherwise noted.

### 2.4. Quantification of EcoRI Activity

To quantify EcoRI activity after incubation of a reaction mixture, digested pCRII-TOPO-OR23 plasmid DNA was examined via agarose gel electrophoresis. We first prepared a 25 mL solution of 1.0% agarose (Nippon Gene) in TAE (Tris-acetate-EDTA) buffer (Promega, Madison, WI, USA) to produce an agarose gel. An ethidium bromide solution (0.5 μL of 10 mg/mL) (Promega) was added before gelation. After incubation with EcoRI, the fluorescence intensity of the band of the excised OR23 fragment was measured via an ImageQuant LAS4000 (GE Healthcare Japan, Tokyo, Japan) with the following conditions: “Exposure Type”: Precision; “Exposure Time”: Manual 1/8 sec; “Sensitivity/Resolution”: Standard; and “Fluorescent Method”: Epi illumination; EtBr; UV (Trans UV) light; 6-5DF50 Filter; and F2.8 Iris.

### 2.5. Statistics

For statistical analyses, Student’s *t*-test (unpaired and bi-sided), Dunnett’s test, and Pearson correlation analysis were performed via Microsoft Excel (Office 365) and JSTAT (Yokohama, Japan). The Dunnett’s test was performed for each experimental group that was examined simultaneously in the laboratory, in comparison to the “no peptide” control group that was examined simultaneously. The repeated measure mode of the Dunnett’s test was used on the assumption that there was no interaction between samples.

## 3. Results

### 3.1. Initially Chosen Seven Peptides

**Figure 3:**
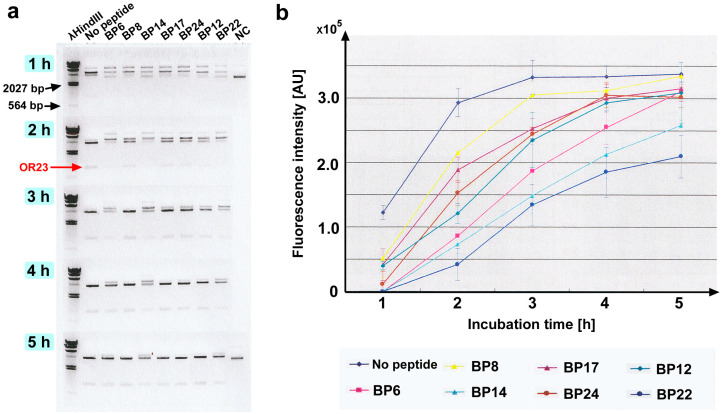
Results of initially chosen seven peptides. (**a**) Agarose gel electrophoresis sampled every hour until 5 h. NC: negative control without EcoRI (shown only at 1 h and 5 h). Excised OR23 bands are indicated. (**b**) Fluorescence intensity versus incubation time. Mean values (±standard deviation) (*n* = 3) are shown. Fluorescence intensity is shown in arbitrary unit (AU).

**Figure 4:**
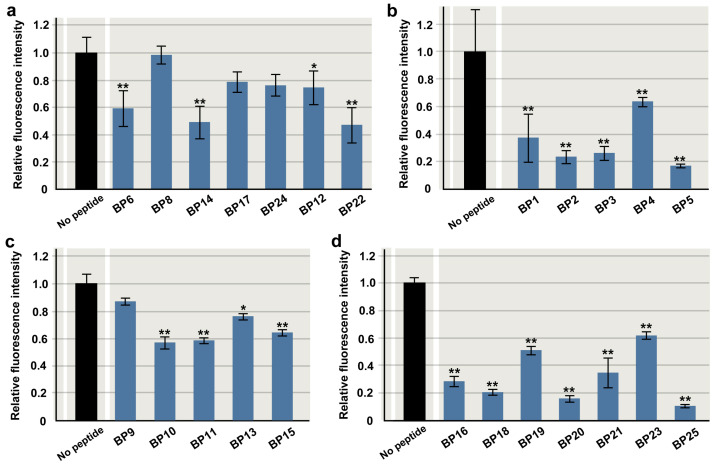
Relative fluorescence intensity of original peptides (except BP7). Mean values (±standard deviation) are shown (*n* = 3 for each peptide). *: *p* < 0.05, **: *p* < 0.01 (Dunnett’s test by comparison to “no peptide”). (**a**) Initially chosen seven peptides. (**b**) N-terminus five peptides. (**c**,**d**) Remaining 13 peptides.

**Figure 5:**
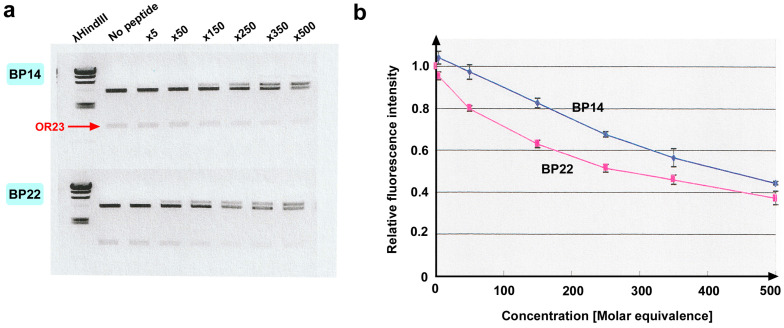
Concentration dependence of inhibitory effects of BP14 and BP22. (**a**) Agarose gel electrophoresis. Concentration (×5 to ×500) indicates molar equivalence to EcoRI. Excised OR23 bands are indicated. (**b**) Relative fluorescence intensity plotted against concentration (molar equivalence). Mean values (±standard deviation) (*n* = 3) are shown.

Among the original 25 blocking peptides (BPs) covering the entire EcoRI protein chain roughly on the basis of secondary structures, seven peptides were arbitrarily chosen to test the feasibility of the experimental conditions for the current PIA: BP6, BP8, BP12, BP14, BP17, BP22, and BP24 (Table 1, Figure 2). These seven peptides were incubated with EcoRI (at the 1-to-500 molar ratio) and the plasmid substrate containing an OR23 insert flanked by two EcoRI recognition sites (GAATTC) for 5 h. As expected, the OR23 insert was excised to obtain DNA fragments of the expected size over time via agarose gel electrophoresis (Figure 3a; Supplemenary Figure S1). The fluorescence intensity of the DNA fragments was plotted against the incubation time (Figure 3b). Compared with the control sample (no peptide), most peptides appeared to have a competitive inhibitory effect by delaying the catalytic rate and did not seem to change the saturation level. The inhibitory effects of these peptides varied; BP14 and BP22 had relatively high inhibitory effects, whereas BP8 had relatively low inhibitory effects. Because the control sample (no peptide) reached a plateau phase at 3 h (Figure 3b), data points at 3 h were used to statistically evaluate the inhibition of EcoRI activity. Among the seven peptides, BP6, BP14, BP12, and BP22 significantly inhibited EcoRI activity (Figure 4a). BP14 for α4, BP22 for α6, and BP6 for βi and α3 were especially notable. Among these peptides, two peptides, BP14 and BP22, were tested for their concentration dependence of the inhibitory effect at a fixed time point (3 h). As expected, the inhibitory effect was demonstrated to be concentration dependent for both peptides (Figure 5a,b; Supplementary Figure S2).

Overall, these results demonstrated that PIA worked on EcoRI under the current conditions using peptides covering secondary structures with an incubation time of 3 h, likely through competitive structural inhibition of EcoRI in a concentration-dependent manner. These results also suggest that reasonably inhibitable sites may be located in many regions of EcoRI. The rest of the experiments were conducted at a 1:500 molar ratio at the fixed time point of 3 h.

### 3.2. Amino-Terminus Five Peptides

The N-terminal region of EcoRI contains an intrinsically disordered loop, and its crystal structure has not been reported. After this region, α1, α2, and β1 are present. It would be interesting to examine whether inhibitory peptides work to inhibit the function of the N-terminal region of EcoRI. Here, five blocking peptides (BPs) covering the N-terminus of the EcoRI sequence were tested: BP1, BP2, BP3, BP4, and BP5 (Table 1, Figure 2). All five peptides were significantly inhibitory (Figure 4b), but BP5 for β1, BP2 for the region before α1, and BP3 for α1 were highly inhibitory. BP1 for the loop for dimerization was also reasonably inhibitory. These results suggest the functionality of the N-terminal region of EcoRI in enzymatic activity and that the N-terminal region of EcoRI may be reasonably targeted in PIA.

### 3.3. Other 13 Peptides to Cover the Entire Protein Chain

The remaining 13 original blocking peptides (BPs) that cover the entire chain based roughly on secondary structures were tested here: BP7, BP9, BP10, BP11, BP13, BP15, BP16, BP18, BP19, BP20, BP21, BP23, and BP25 (Table 1). Most peptides were significantly inhibitory, although the effects varied among the peptides (Figure 4c,d). BP25 and BP20 for the C-terminal region and BP18 for α5 were especially notable (Figure 4d), suggesting that they may be structurally and functionally important. BP16 in the region between β4 and βiii was also highly inhibitory.

### 3.4. Randomized Peptides with the Same Amino Acid Contents

**Figure 6:**
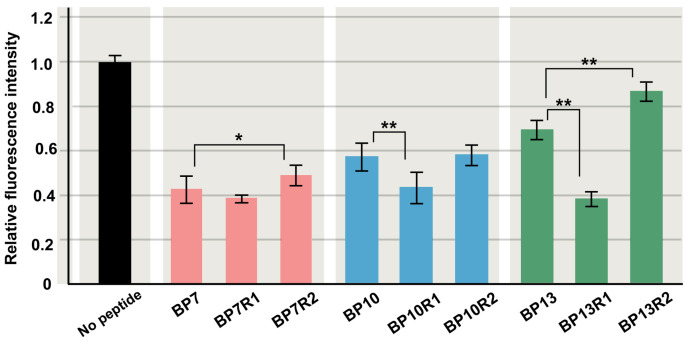
Relative fluorescence intensity of randomized peptides of BP7, BP10, and BP13. Two randomized peptides from each original peptide were tested. Mean values (±standard deviation) are shown (*n* = 3 for each peptide). *: *p* < 0.05, **: *p* < 0.01 (Student’s *t*-test by comparison to each endogenous BP).

Here, to test the sequence specificity of blocking peptides (BPs), the amino acid sequences of BP7, BP10, and BP13 were randomized to produce new peptides (Table 2). Two randomized peptides for each of BP7, BP10, and BP13 were synthesized, namely, BP7R1, BP7R2, BP10R1, BP10R2, BP13R1, and BP13R2. Compared with the original peptides, two of these randomized peptides (BP7R2 and BP13R2) were significantly less inhibitory, but unexpectedly, two of them (BP10R1 and BP13R1) were significantly more inhibitory (Figure 6). Two of them (BP7R1 and BP10R2) were as inhibitory as the original peptides. These results suggest that the presumed competitive interactions with EcoRI are not necessarily sequence-specific and that amino acid contents may be important, although these results and interpretations are not consistent with other results and interpretations.

### 3.5. Additional 22 Staggered Peptides

**Figure 7:**
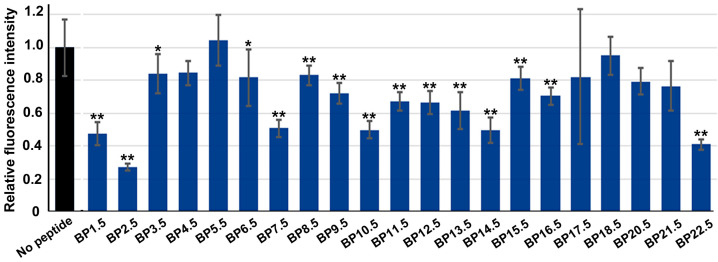
Relative fluorescence intensity of staggered peptides. BP19.5 is not included in this figure. Mean values (±standard deviation) are shown (*n* = 3 for each peptide). *: *p* < 0.05, **: *p* < 0.01 (Dunnett’s test by comparison to “no peptide”).

Although the original 25 blocking peptides used thus far were designed to cover the entire chain of the EcoRI protein, the peptide windows may bias the results. To circumvent this potential problem, we synthesized 22 additional staggered blocking peptides (BPs) (Table 3). This group of peptides was designed so that their central amino acids were located at the gap between the two consecutive original peptides. Thus, they contained the C-terminal and N-terminal regions of the two consecutive peptides. These staggered peptides were named BP1.5, BP2.5, BP3.5, and so on. For example, BP1.5 is composed of the C-terminal sequence of BP1 and the N-terminal sequence of BP2. Many of these peptides were significantly inhibitory, although to various degrees (Figure 7). Among them, BP2.5 for α1 was notable.

### 3.6. Nonspecific Nuclease Activity with BP19.5

In the analysis of the reaction product with BP19.5, no DNA fragment or plasmid DNA was detected via agarose gel electrophoresis, and the fluorescence intensity values were not measurable (*n* = 3). This reaction mixture containing EcoRI and BP19.5 appeared to degrade the plasmid DNA entirely. No other peptide showed this unexpected activity. The DNA concentration at 3 h was 3.297 ± 0.006 ng/μL without any peptide in the reaction mixture and 0.332 ± 0.003 ng/μL with BP19.5, indicating a statistically significant difference (*n* = 3; unpaired bi-sided Student’s *t*-test; *p* = 1.6 × 10^−11^). Two deletion peptides of BP19.5, namely, BP19.5N and BP19.5C (Table 4), were also tested, and no DNA was detected in agarose gel electrophoresis in both peptides (*n* = 3). Therefore, BP19.5 and its derivatives are likely to cause EcoRI to become a nonspecific nuclease. In other words, BP19.5 was likely a specificity/activity modifier of EcoRI instead of an inhibitor.

### 3.7. Short Peptides

Thus far, peptide length has been set arbitrarily from 10 to 14 aa in this study. However, shorter peptides might be sufficient to inhibit EcoRI activity. Three short peptides (6 aa) were synthesized on the basis of an original peptide. For example, short peptides were named BP6N (the N-terminal sequence of BP6), BP6M (the middle sequence of BP6), and BP6C (the C-terminal sequence of BP6). The N, M, and C peptides were synthesized based on the original ten peptides (BP6, BP7, BP9, BP10, BP11, BP12, BP13, BP14, BP15, and BP22) (Table 5).

**Figure 8:**
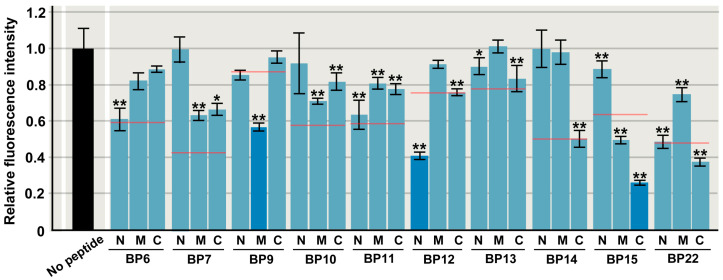
Relative fluorescence intensity of short peptides. N, M, and C indicate N-terminal, middle, and C-terminal sequences of original peptide, respectively. Mean values (±standard deviation) are shown (*n* = 3 for each peptide). Red lines indicate relative fluorescence intensity values of original peptides. BP9M, BP12N, and BP15C are shown as dark blue bars to indicate a substantial decrease in relative fluorescence intensity values of original peptides. *: *p* < 0.05, **: *p* < 0.01 (Dunnett’s test by comparison to “no peptide”).

The inhibitory effects of a set of three peptides varied (Figure 8). For example, in the case of BP6, the inhibitory effect was stronger in the N-terminal portion, BP6N, and it was as inhibitory as the original BP6, indicating that the core of the inhibitory sequence is located at the N-terminal portion of BP6, corresponding to βi. Similarly, in the case of BP14, the inhibitory effect was stronger in the C-terminal portion, BP14C, and it was as inhibitory as the original BP14, indicating that the core of the inhibitory sequence is located at the C-terminal portion of BP14, corresponding to α4. On the other hand, in the case of BP15, the inhibitory effect was more intense in the C-terminal portion, BP15C, and it was more inhibitory than the original BP15, corresponding to β4. Similarly, BP12N corresponding to αi and BP9M corresponding to β2 were more inhibitory than the original BP12 and BP9, respectively, indicating the positions of the target sequences. In contrast, in the case of BP7 and BP10, all three short peptides were less inhibitory than the original BP7 and BP10, suggesting that the full length of the original peptides is required to maintain the original level of inhibition. These results suggest that inhibitory functions are found in smaller portions of the original peptides in some cases, but in other cases, longer peptides are more inhibitory than shorter peptides.

### 3.8. Overview of the Inhibitory Effects

**Figure 9:**
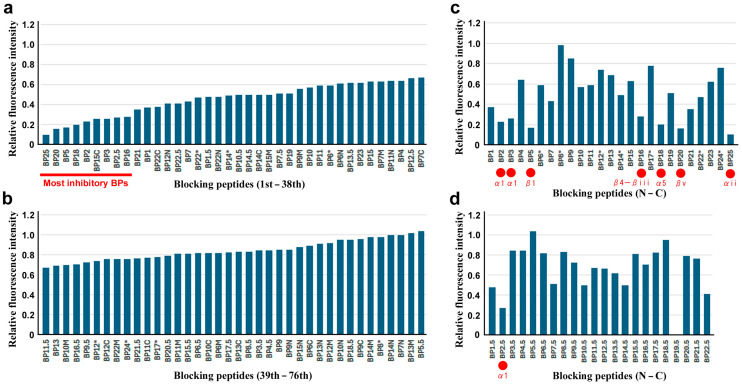
Overview of inhibitory effects. Asterisks indicate initially chosen seven BPs. (**a**) Ranks 1st to 36th. The nine most inhibitory BPs are indicated. (**b**) Rank 37th to 72nd. (**c**) Original BP series ordered from the N-terminal to the C-terminal regions. The seven most inhibitory BPs in (**a**) are indicated by red circles. (**d**) Staggered BP series ordered from N-terminal to C-terminal regions. One of the most inhibitory BPs in (**a**) is indicated by a red circle. This graph shows the same results as Figure 7 but was aligned with (**c**) for visual comparison. BP19.5 did not show any fluorescence intensity of the DNA fragment.

Here, the overall tendencies of the inhibitory effects of BPs were examined. When the peptides were placed in the order of relative fluorescence intensity, the inhibitory effects varied from BP25 (most inhibitory) (Figure 9a) to BP5.5 (least inhibitory) (Figure 9b). There seemed to be no critical threshold of the inhibitory levels, but nine BPs with relative fluorescence intensities less than 0.3 (BP25, BP20, BP5, BP18, BP2, BP15C, BP3, BP2.5, and BP16) may be considered promising as short constituent sequences of important EcoRI functional sites and as drug targets (Figure 9a). Notably, only one staggered peptide (BP2.5) and one short peptide (BP15C) were categorized into the nine most effective peptides. The other seven peptides among the most effective peptides were all from the original peptides. These results suggest that peptide design in accordance with secondary structures may be more effective and that short peptides may not be effective in the inhibition of EcoRI activity, although there are a few exceptions. As presented in Figure 8, BP15C, BP12N, and BP9M were shorter but more inhibitory than the original peptides were.

When the original peptides (Figure 9c) and the staggered peptides (Figure 9d) were ordered from the N-terminal to C-terminal regions, the most effective peptides were located in the N-terminal region (BP2, BP2.5, BP3, and BP5), in the C-terminal region (BP25), and in the middle region (BP16, BP 18, and BP20). In contrast, a large middle portion of the EcoRI region from BP6 to BP15 was relatively ineffective, although the major active sites are located in this region.

### 3.9. Potential Correlations Among Physicochemical Factors

**Figure 10:**
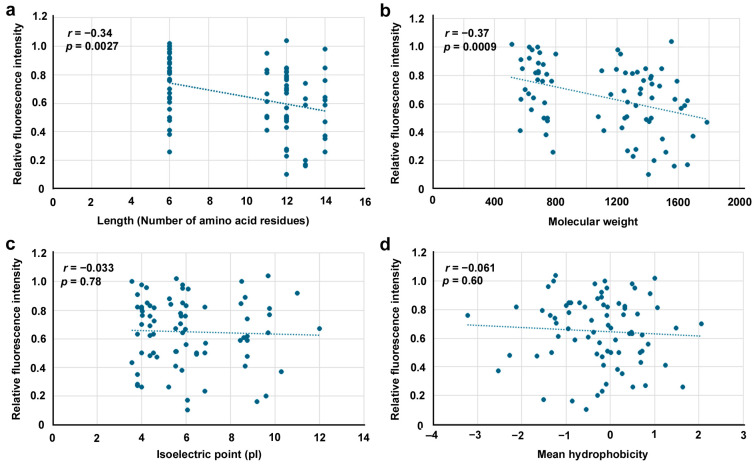
Scatter plots of four physicochemical factors of 76 BPs used in this study. Pearson correlation coefficients and their associated *p* values are shown. (**a**) Peptide length (number of amino acid residues). (**b**) Molecular weight. (**c**) Isoelectric point (pI). (**d**) Mean hydrophobicity.

**Figure 11:**
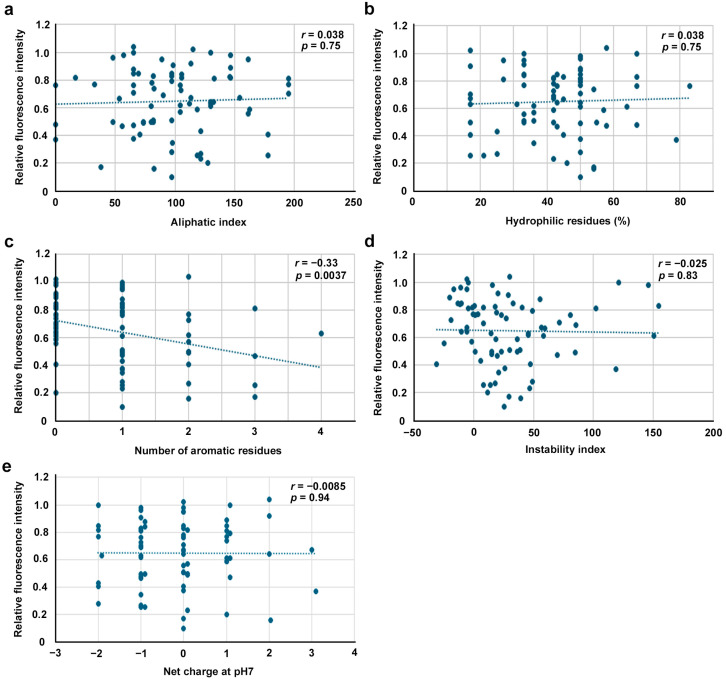
Scatter plots of additional five physicochemical factors of 76 BPs used in this study. Pearson correlation coefficients and their associated *p* values are shown. (**a**) Aliphatic index. (**b**) Hydrophilic residues (%). (**c**) Number of aromatic residues. (**d**) Instability index. (**e**) Net charge at pH7.

Here, potential correlations between the inhibitory effects and physicochemical factors of the peptides via data from 76 BPs (excluding BP19.5, BP19.5N and BP19.5C) were examined, namely, length (the number of amino acid residues), molecular weight, isoelectric point, and mean hydrophobicity (Figure 10). Low but significant correlations were observed for length (Figure 10a) and molecular weight (Figure 10b), and no significant correlations were observed for the isoelectric point (Figure 10c) or mean hydrophobicity (Figure 10d). Additional five physicochemical factors were also examined, namely, aliphatic index, the percentage of hydrophilic residues, the number of aromatic residues, instability index, and net charge at pH7 (Figure 11). Among them, low but significant correlation was observed for the number of aromatic residues (Figure 11c). Other factors did not show significant correlations (Figure 11a–e).

## 4. Discussion

In the present study, we focused on a model protein, EcoRI, to evaluate the feasibility of PIA to characterize functionally important accessible sites in a protein chain. The original 25 blocking peptides (BPs) were designed in reference to secondary structures to cover the entire protein chain without critical information on functional sites. In this way, information on secondary structures is helpful for designing BPs, but PIA can be performed without such information. In fact, PIA does not need any three-dimensional information. Intrinsically disordered regions can also be examined. Moreover, PIA can exhaustively examine the entire sequence of a protein chain. Thus, PIA can be considered a hypothesis-free test. Alternatively, PIA can be used in accordance with a hypothesis on the basis of previous PIA results or other information on functional sites. The PIA results are likely influenced by the accessibility of the peptides to the corresponding sequences and cannot completely identify “functional” sites in a strict sense. This also means that the PIA results reflect accessibility automatically, which may be convenient for identifying drug candidates. The discovery of unknown functional sites in a broad sense may be expected in the course of PIA, as shown in the present study. To execute PIA, the reaction conditions for a protein of interest are important. The present study used nonoptimum reaction buffer, an incubation time of 3 h, and an arbitrary 1:500 concentration of BPs on the basis of the initial assessment. Such a search for reaction conditions is important for PIA to work properly.

Among the 79 endogenous peptides tested in the present study, the original set of peptides worked better than the staggered peptides and short peptides did, demonstrating that secondary structures are indeed functional units of intra- and intermolecular interactions and may be considered recommended units of BP design. Indeed, the nine most efficient peptides largely corresponded to secondary structures (Figure 9). An exception was BP16, which is located between β4 and βiii (Figure 2). Site-directed mutagenesis studies have revealed several amino acid residues in the middle region of EcoRI for catalytic function or DNA recognition [64,65,66,67,68,69,70,71,72,73,74,75,76,77], but this region was largely not inhibitory in the present study. The major active site, which is composed of α4 and other secondary structures, was not inhibited very much by BPs, possibly because of low accessibility. These results may challenge the conventional view of rational drug design that mainly targets active sites. Rather, according to the present study, functional sites that differ from active sites may be promising for the development of novel peptide drugs.

Randomized peptides of BP7, BP10, and BP13 showed mixed results: BP7R2 and BP13R showed lower inhibition (higher EcoRI activity) than the original peptides did, which is theoretically expected if the inhibition is sequence specific, but BP10R1 and BP13R1 showed greater inhibition, which is theoretically unexpected. The reason for these results is not clear, but these perplexing results suggest that the inhibitory effect may not be completely sequence-specific and that amino acid contents are also important, at least in these three cases. Correlation analysis revealed the importance of aromatic residues (F, H, W, and Y) for inhibitory effects, supporting the idea that amino acid contents are also important. Surely, other results suggest the importance of sequence specificity. The validity and interpretation of these data should be clarified in the future.

Although the results of the randomized peptides were difficult to interpret, this experiment is an example of a “mutagenesis” experiment for functional sites. In addition to randomized sequences, many types of mutagenesis experiments are readily possible in PIA: point mutations, domain swapping, and indels. Longer or shorter peptides of various sequences corresponding to protein sequences are possible. In designing blocking peptides, the size and position of windows for short constituent sequences (SCSs) to be covered by peptides may be changed at the discretion of researchers. For example, to perform an exhaustive scan, an SCS window in a protein chain can be slid one at a time from the N-terminus to the C-terminus. Staggered peptides are not necessary in that case, making the PIA truly hypothesis-free in terms of the position of functional sites. To analyze protein activity, the present study used the fluorescence intensity of DNA fragments as output data, but in the future, other methods that can be measured in a high-throughput manner, such as colorimetry based on ELISA (enzyme-linked immunosorbent assay), are favored. Peptide arrays for PIA may be challenging because of the low affinity of BPs for the target protein. Combinatorial addition of two or more peptides in a reaction mixture at the same time may hasten the assay to narrow down the candidate inhibitors.

The short peptides designed from the original peptides used in the present study are indeed like deletion mutants and might have been too short to be effective as an inhibitor of EcoRI. These peptides may be too short to mimic the corresponding secondary structures or bind stably to the target sites, although an exception was BP15C, which was more inhibitory than the original BP15 peptide. In accordance with this view, correlation analysis revealed that the number of amino acid residues (peptide length) and molecular weight were negatively correlated with the relative fluorescence intensity. In other words, peptide length and molecular weight were positively correlated with the inhibitory effects of BPs. Peptide length and molecular weight are directly proportional to approximate volume of peptides [88]. These results are theoretically expected, considering that the inhibition process is sequence-specific and structure-specific but may contradict the unexpected results of the randomized peptides. On the other hand, the number of aromatic residues (F, H, W, and Y) was negatively correlated with the relative fluorescence intensity. In other words, the number of aromatic residues was positively correlated with the inhibitory effects of BPs, suggesting the importance of amino acid contents. In contrast, the isoelectric point, mean hydrophobicity, aliphatic index, the percentage of hydrophilic residues, instability index, and net charge at pH7 were not correlated. It is interesting to investigate the role of aromatic rings in the inhibition process in the future.

Unexpected results were also obtained in BP19.5, which probably induced nonspecific nuclease activity of EcoRI. Alternatively, BP19.5 itself may have nuclease activity, but this is unlikely because the amino acid sequence of EcoRI corresponding to BP19.5 is not known to have nuclease activity. BP19.5 contains a cysteine residue (C218), which may form an intermolecular disulfide bond and could contribute to nonspecific nuclease activity of EcoRI. However, both BP19.5N (not containing C218) and BP19.5C (containing C218) induced such nuclease activity, suggesting no direct contribution of C218 to the nonspecific nuclease induction. Similarly, BP20 also contains C218 but does not induce nuclease activity, although BP20 has a strong inhibitory effect. Rather, N216 and L217 together with their either N-terminal or C-terminal regions may play an important role in the nonspecific nuclease induction because these two residues were contained in both BP19.5N and BP19.5C, although BP19 did not have such an induction ability. In any case, BP19.5 can be considered a specificity/activity modifier of EcoRI instead of an inhibitor. These unexpected results are perplexing but may lead to unexpected discoveries, which could be considered advantages of the hypothesis-free analysis of PIA.

Mechanistically, if BP19.5 does not bind to substrate DNA and degrade it nonspecifically, there was no type I inhibitory peptide in the present study. Most BPs are likely type II inhibitory peptides. Among these type II inhibitory peptides, the possible active site inhibitors, including Mg^2+^-binding sites, were BP8, BP8.5, BP9, BP10, BP10.5, BP13, BP12.5, BP13.5, BP17, BP17.5, BP18, and BP18.5, according to Heitman (1992) [55]. Except for BP18, these BPs were not very inhibitory. The peptides of the possible dimerization inhibitors were BP1, BP1.5, BP2, BP6, BP6.5, BP7, BP7.5, BP8, BP8.5, BP20, BP20.5, and BP21. They may be classified into Type III inhibitory peptides or PPI inhibitors. Notably, the intrinsically disordered N-terminal region was inhibited by BP1 and its adjacent BP2, which is consistent with the view that intrinsically disordered portions can be targeted for novel drugs [89,90]. Despite various levels of inhibition, it is surprising that many peptides were more or less inhibitory. The noninhibitory peptides included BP8, BP5.5, BP18.5, BP7N, BP9C, BP10N, BP13M, BP14N, and BP14M.

The most effective BPs were BP25, BP20, BP5, BP18, BP2, BP15C, BP3, BP25, and BP16 (Figure 9). Among these nine BPs, BP18 covers α5, a part of the active site. This is the only exception, among the nine most effective BPs, that correspond to the active site, although it is still outside the major active sites (βii-β2 and β3). BP20 and BP2 likely inhibit dimerization sites, as shown in the solution structure studies of Watrob et al. (2001) [78], as type III inhibitors. These are PPI inhibitors, demonstrating the feasibility of using PPIs as drug targets. In contrast, the PPI inhibitor that has been examined corresponding to α4 in a previous study (BP14 in the present study) [81] showed only a moderate inhibitory effect. The other BPs among the nine most effective peptides may work as type II inhibitors, which have not been predicted in previous studies.

To discuss individual BPs with high inhibition, any of the three consecutive peptides (BP2, BP2.5, and BP3), but not their flanking peptides (BP1.5 and BP3.5), worked as α1 inhibitors. These three peptides share the VIGI sequence, which may be the core target of functional inhibition. In contrast, the highly inhibitory effect of BP5 disappeared in its flanking peptides, BP4.5 and BP5.5, suggesting that the central S54 in BP5 may be the core target of BP5-mediated inhibition. Likewise, the highly inhibitory effect of BP16 was not observed in the flanking BP15.5 and BP16.5 regions, suggesting that the loop region between β4 and βiii (GSNFLTE) instead of secondary structures may be an important region for BP16-mediated inhibition. The highly inhibitory effect of BP18 was not observed in the flanking BP17.5 and BP18.5, suggesting the functional importance of the N-terminal portion of α5 and its connecting loop, which contains previously identified functional sites (I197, R200 and R203). In the case of BP20, which showed high inhibition, the flanking BP19 and BP20.5 did not show high inhibition, and BP19.5 was an inducer of nuclease activity. Thus, not only βv but also its N-terminal and C-terminal amino acid residues may be important for BP20-mediated inhibition. Finally, for high inhibition by BP25, the full length of αii appears to be required because BP24 did not work well. Together, many of these potential “functional” sites are newly identified, and they may be examined further with additional BPs and with other methods, such as site-directed mutagenesis, in the future, potentially leading to the development of novel drugs. Alternatively, the identified sites may be used for raising neutralizing antibodies as antigenic peptide epitopes. In that case, the accessibility of target regions for antibodies may be high because PIA requires high accessibility of target regions for BPs.

An important question is how some of these BPs can be modified chemically to make protein‒peptide interactions more specific and more stable. To do so, various chemical modifications of peptides may be tested on a trial-and-error basis, but realizing stable secondary structures in peptides may be an important direction for a chemical modification strategy. Replacing peptide bonds with stable nonpeptide structures may be performed as peptidomimetics. EcoRI inhibitors may not be useful drugs in terms of experimental and clinical applications. However, similar strategies involving human or pathogenic proteins may have potential clinical applications. For example, the spike protein of SARS-CoV-2, which has been already analyzed based on SCS distributions [91,92,93], may be subjected to PIA for novel drugs against SARS-CoV-2 infection. SCS-based epitopes of spike proteins for effective neutralizing antibodies may also be identified with the help of PIA. Indeed, peptides and peptidomimetics have been considered excellent candidates for novel drugs for COVID-19 treatment [94,95,96]. Proteins from experimental organisms may also be helpful for understanding the molecular functions of target proteins. For clinical use, peptide inhibitors should be chemically stable and well-tested for side effects. In contrast, for use in experimental animals, in vivo bioassays may be performed directly using relatively high concentrations of targeting peptides to knock down a target protein like an antibody. For example, in our previous study, direct protein delivery to butterfly wing epithelial cells appeared to be more efficient for smaller proteins [97,98]. Peptides may be administered instead of antibodies in vivo for direct delivery to cells, which may lead to a novel method for studying protein functions in vivo.

## Figures and Tables

**Table 1:** Original 25 blocking peptides (BPs).

Name	Amino Acid Number	Amino Acid Sequence	Length (aa)	Molecular Weight	Isoelectric pI	Mean Hydrophobicity
BP1	2–15	SNKKQSNRLTEQHK	14 aa	1697.87	10.29	−2.529
BP2	12–23	EQHKLSQGVIGI	12 aa	1308.50	6.85	−0.183
BP3	20–33	VIGIFGDYAKAHDL	14 aa	1518.73	5.21	0.514
BP4	34–47	AVGEVSKLVKKALS	14 aa	1428.74	9.7	0.471
BP5	48–60	NEYPQLAFRYRDS	13 aa	1658.79	6.07	−1.508
BP6 *	61–74	IKKTEINEALKKID	14 aa	1642.96	8.43	−0.800
BP7	73–84	IDPDLGGTLFVS	12 aa	1233.38	3.56	0.683
BP8 *	82–93	FVSNSSIKPDGG	12 aa	1207.31	5.84	−0.350
BP9	89–102	KPDGGIVEVKDDYG	14 aa	1491.62	4.23	−0.929
BP10	103–116	EWRVVLVAEAKHQG	14 aa	1621.86	6.86	−0.243
BP11	116–127	GKDIINIRNGLL	12 aa	1325.57	8.75	0.117
BP12 *	123–135	RNGLLVGKRGDQD	13 aa	1427.58	8.75	−1.254
BP13	135–146	DLMAAGNAIERS	12 aa	1247.39	4.37	−0.050
BP14 *	146–157	SHKNISEIANFM	12 aa	1390.58	6.47	−0.308
BP15	158–170	LSESHFPYVLFLE	13 aa	1580.80	4.51	0.500
BP16	170–181	EGSNFLTENISI	12 aa	1323.42	3.80	−0.092
BP17 *	182–193	TRPDGRVVNLEY	12 aa	1418.57	5.74	−0.942
BP18	194–206	NSGILNRLDRLTA	13 aa	1442.64	9.60	−0.285
BP19	206–217	AANYGMPINSNL	12 aa	1264.42	5.57	−0.067
BP20	216–228	NLCINKFVNHKDK	13 aa	1572.85	9.20	−0.854
BP21	229–242	SIMLQAASIYTQGD	14 aa	1497.68	3.80	0.271
BP22 *	243–252	GREWDSKIMFEIMF	14 aa	1789.10	4.68	−0.186
BP23	252–265	FEIMFDISTTSLRV	14 aa	1658.93	4.37	0.714
BP24 *	259–272	STTSLRVLGRDLFE	14 aa	1593.80	5.79	−0.071
BP25	266–277	LGRDLFEQLTSK	12 aa	1406.60	6.07	−0.550

* Initially chosen seven peptides.

**Table 2:** Randomized peptides from original BP7, BP10 and BP13 sequences.

Name	Amino Acid Number	Amino Acid Sequence	Length (aa)	Molecular Weight	Isoelectric pI	Mean Hydrophobicity
BP7R1	n.a.	VGLILSTGDFDP	12 aa	1233.38	3.56	0.683
BP7R2	n.a.	DGLTPFSDVLGI	12 aa	1233.38	3.56	0.683
BP10R1	n.a.	VEGQELAVKAHRWV	14 aa	1621.86	6.73	−0.243
BP10R2	n.a.	AGRKQEVHAVWVEL	14 aa	1621.86	6.80	−0.243
BP13R1	n.a.	EALDIAMGRNSA	12 aa	1247.39	4.37	−0.050
BP13R2	n.a.	LAMRGASIADEN	12 aa	1247.39	4.37	−0.050

n.a.: not applicable.

**Table 3:** Additional 22 staggered blocking peptides (BPs).

Name	Amino Acid Number	Amino Acid Sequence	Length (aa)	Molecular Weight	Isoelectric pI	Mean Hydrophobicity
BP1.5	8–19	NRLTEQHKLSQG	12 aa	1410.55	8.75	−1.658
BP2.5	16–27	LSQGVIGIFGDY	12 aa	1268.43	3.80	0.792
BP3.5	28–39	AKAHDLAVGEVS	12 aa	1196.33	5.32	0.192
BP4.5	42–52	VKKALSNEYPQL	12 aa	1389.61	8.47	−0.700
BP5.5	55–66	FRYRDSIKKTEI	12 aa	1555.80	9.70	−1.233
BP6.5	65–79	EALKKIDPDLGG	12 aa	1255.43	4.56	−0.567
BP7.5	78–88	GGTLFVSNSSI	11 aa	1081.19	5.52	0.718
BP8.5	86–96	SSIKPDGGIVE	11 aa	1101.22	4.37	−0.155
BP9.5	97–109	VKDDYGEWRVVL	12 aa	1478.67	4.56	−0.425
BP10.5	111–121	EAKHQGKDIIN	11 aa	1252.39	6.85	−1.327
BP11.5	120–131	INIRNGLLVGKR	12 aa	1352.65	12.01	0.008
BP12.5	130–140	KRGDQDLMAAG	11 aa	1161.30	5.96	−0.945
BP13.5	141–151	NAIERSHKNIS	11 aa	1268.40	8.75	−1.173
BP14.5	152–163	EIANFMLSESHF	12 aa	1424.59	4.51	0.192
BP15.5	165–175	YVLFLEGSNFL	11 aa	1301.50	4.00	1.064
BP16.5	176–187	TENISITRPDGR	12 aa	1358.47	5.74	−1.225
BP17.5	188–199	VVNLEYNSGILN	12 aa	1334.49	4.00	0.333
BP18.5	201–211	LDRLTAANYGM	11 aa	1224.40	5.84	−0.073
BP19.5	223–234	PINSNLCINK	10 aa	1383.63	8.57	−0.508
BP20.5	237–252	VNHKDKSIMLQA	12 aa	1426.46	4.03	−1.542
BP21.5	246–257	IYTQGDGREWDS	12 aa	1375.62	4.03	0.291
BP22.5	212–221	DSKIMFEIMFD	11 aa	1115.31	8.64	−0.150

**Table 4:** Deletion peptides from BP19.5.

Name	Amino Acid Number	Amino Acid Sequence	Length (aa)	Molecular Weight	Isoelectric pI	Mean Hydrophobicity
BP19.5N	212–217	PINSNL	6 aa	656.74	5.96	−0.183
BP19.5C	216–221	NLCINK	6 aa	703.85	8.22	−0.017

**Table 5:** Short blocking peptides (BPs) from original 10 peptides.

Name	Amino Acid Number	Amino Acid Sequence	Length (aa)	Molecular Weight	Isoelectric pI	Mean Hydrophobicity
BP6N	61–66	IKKTEI	6 aa	730.90	8.59	−0.500
BP6M	65–70	EINEAL	6 aa	687.75	3.80	−0.067
BP6C	69–74	ALKKID	6 aa	686.85	8.64	−0.200
BP7N	73–78	IDPDLG	6 aa	628.68	3.56	−0.117
BP7M	76–81	DLGGTL	6 aa	574.63	3.80	0.433
BP7C	79–84	GTLFVS	6 aa	622.72	5.52	1.483
BP9N	89–94	KPDGGI	6 aa	585.66	5.84	−0.883
BP9M	93–98	GIVEVK	6 aa	643.78	6.00	0.850
BP9C	97–102	VKDDYG	6 aa	695.73	4.21	−1.400
BP10N	103–108	EWRVVL	6 aa	800.96	6.10	0.550
BP10M	107–112	VLVAEA	6 aa	600.71	4.00	2.050
BP10C	111–116	EAKHQG	6 aa	668.71	6.85	−2.117
BP11N	116–121	GKDIIN	6 aa	658.75	5.84	−0.383
BP11M	119–124	IINIRN	6 aa	741.89	9.75	0.333
BP11C	121–127	IRNGLL	6 aa	684.84	9.75	0.617
BP12N	124–129	NGLLVG	6 aa	571.67	5.52	1.250
BP12M	126–132	LVGKRG	6 aa	628.77	11.00	−0.200
BP12C	130–135	KRGDQD	6 aa	717.74	5.96	−3.217
BP13N	135–140	DLMAAG	6 aa	576.67	3.80	0.900
BP13M	138–143	AAGNAI	6 aa	515.57	5.57	1.000
BP13C	141–146	NAIERS	6 aa	688.74	6.00	−1.000
BP14N	146–151	SHKNIS	6 aa	684.75	8.49	−1.283
BP14M	149–154	NISEIA	6 aa	645.71	4.00	0.500
BP14C	152–157	EIANFM	6 aa	723.84	4.00	0.667
BP15N	158–163	LSESHF	6 aa	718.76	5.24	−0.283
BP15M	161–166	SHFPYV	6 aa	748.84	6.46	0.017
BP15C	165–170	YVLFLE	6 aa	782.93	4.00	1.633
BP22N	243–248	GREWDS	6 aa	748.75	4.37	−2.267
BP22M	245–250	EWDSKI	6 aa	776.84	4.37	−1.350
BP22C	247–252	DSKIMF	6 aa	739.88	5.80	0.167

**Table 6:** Additional physicochemical parameters of blocking peptides (BPs).

Name	Aliphatic Index	Hydrophilic Residues (%)	Number of Aromatic Residues	Instability Index	Net Charge at pH7
BP1	27.86	79	1	118.93	3.09
BP2	121.67	42	1	46.85	0.09
BP3	118.57	21	3	14.07	−0.91
BP4	132.14	43	0	−10.29	2.00
BP5	37.69	54	3	29.55	0.00
BP6	118.57	57	0	18.61	1.00
BP7	121.67	25	1	6.09	−2.00
BP8	56.67	50	1	15.86	0.00
BP9	69.29	43	1	−12.92	−2.00
BP10	104.29	36	2	−1.45	0.09
BP11	162.50	42	0	36.47	1.00
BP12	82.31	54	0	27.46	1.00
BP13	90.00	42	0	85.61	−1.00
BP14	73.33	50	2	85.09	0.09
BP15	112.31	31	4	45.70	−1.91
BP16	97.50	50	1	49.31	−2.00
BP17	80.83	42	1	18.93	0.00
BP18	127.69	46	0	11.72	1.00
BP19	81.67	33	1	38.71	0.00
BP20	82.31	54	2	39.13	2.04
BP21	97.86	36	1	21.12	−1.00
BP22	55.71	43	3	20.00	−1.00
BP23	104.29	36	2	45.61	−1.00
BP24	104.29	43	0	23.04	0.00
BP25	97.50	50	1	25.22	0.00
BP7R1	121.67	25	1	15.07	−2.00
BP7R2	121.67	25	1	18.14	−2.00
BP10R1	104.29	36	2	49.77	0.09
BP10R2	104.29	36	2	33.89	0.09
BP13R1	90.00	42	0	19.45	−1.00
BP13R2	90.00	42	0	−18.34	−1.00
BP1.5	65.00	58	1	69.98	1.09
BP2.5	121.67	25	2	18.14	-1.00
BP3.5	105.83	33	1	−11.27	−0.91
BP4.5	97.50	50	1	33.10	1.00
BP5.5	65.00	58	2	29.87	2.00
BP6.5	105.83	42	0	−0.98	−1.00
BP7.5	97.27	36	1	30.10	0.00
BP8.5	97.27	45	0	0.95	−1.00
BP9.5	105.00	42	2	−19.12	−1.00
BP10.5	80.00	55	1	1.37	0.09
BP11.5	154.17	42	0	56.56	3.00
BP12.5	53.64	45	0	59.74	0.00
BP13.5	80.00	64	1	150.45	1.09
BP14.5	73.33	42	2	36.15	−1.91
BP15.5	132.73	27	3	−4.57	−1.00
BP16.5	65.00	50	0	71.60	0.00
BP17.5	145.83	42	1	18.14	−1.00
BP18.5	89.09	27	1	−5.48	0.00
BP19.5	117.00	50	0	32.68	0.95
BP20.5	97.50	50	1	48.99	1.09
BP21.5	32.50	50	2	−0.67	−2.00
BP22.5	70.91	45	2	47.61	−2.00
BP19.5N	130.00	50	0	8.33	0.00
BP19.5C	130.00	50	0	47.80	0.95
BP6N	130.00	50	0	58.38	1.00
BP6M	146.67	50	0	40.43	−2.00
BP6C	146.67	50	0	−19.97	1.00
BP7N	130.00	33	0	-4.23	−2.00
BP7M	130.00	17	0	14.75	−1.00
BP7C	113.33	17	1	−5.82	0.00
BP9N	65.00	33	0	−10.38	0.00
BP9M	161.67	33	0	−24.77	0.00
BP9C	48.33	50	1	−10.62	−1.00
BP10N	161.67	33	1	−16.72	0.00
BP10M	195.00	17	0	8.33	−1.00
BP10C	16.67	50	1	8.33	0.09
BP11N	130.00	50	0	−5.82	0.00
BP11M	195.00	50	0	102.13	1.00
BP11C	195.00	33	0	3.85	1.00
BP12N	178.33	17	0	−30.87	0.00
BP12M	113.33	33	0	20.22	2.00
BP12C	0.00	83	0	80.62	0.00
BP13N	98.33	17	0	28.90	−1.00
BP13M	115.00	17	0	−5.82	0.00
BP13C	81.67	67	0	154.80	0.00
BP14N	65.00	67	1	121.03	1.09
BP14M	146.67	50	0	145.77	−1.00
BP14C	81.67	33	1	15.38	−1.00
BP15N	65.00	50	1	55.25	−0.91
BP15M	48.33	17	2	23.15	0.09
BP15C	178.33	17	1	8.33	−1.00
BP22N	0.00	67	1	15.38	−1.00
BP22M	65.00	67	1	1.23	−1.00
BP22C	65.00	50	1	26.28	0.00

## Data Availability

The original contributions presented in this study are included in the article. Further inquiries can be directed to the corresponding author.

## Supplementary Materials

The following supporting information can be downloaded at: https://www.mdpi.com/article/10.3390/biotech14010001/s1, Figure S1: Original gel images for Figure 3a; Figure S2: Original gel images for Figure 5a.
