# Supplementary material for: Peptide Inhibitor Assay for Allocating Functionally Important Accessible Sites Throughout a Protein Chain: Restriction Endonuclease EcoRI as a Model Protein System
Source: BioTech (Basel). 2024 Dec 30;14(1):1. doi: 10.3390/biotech14010001 (PMC11755562; doi:10.3390/biotech14010001)
Supplement: Supplementary file 1 [file biotech-14-00001-s001.zip › biotech-3308110-supplementary.pdf]

Peptide Inhibitor Assay for Allocating Functionally Important Accessible Sites Throughout a Protein Chain: Restriction Endonuclease EcoRI as a Model Protein System

Joji M. Otaki

The BCPH Unit of Molecular Physiology, Department of Chemistry, Biology and Marine Science, Faculty of Science, University of the Ryukyus, Nishihara, Okinawa 903-0213, Japan; otaki@sci.u-ryukyu.ac.jp;
Tel.: +81-98-895-8557

Supplementary Figures S1 and S2

Figure 3a

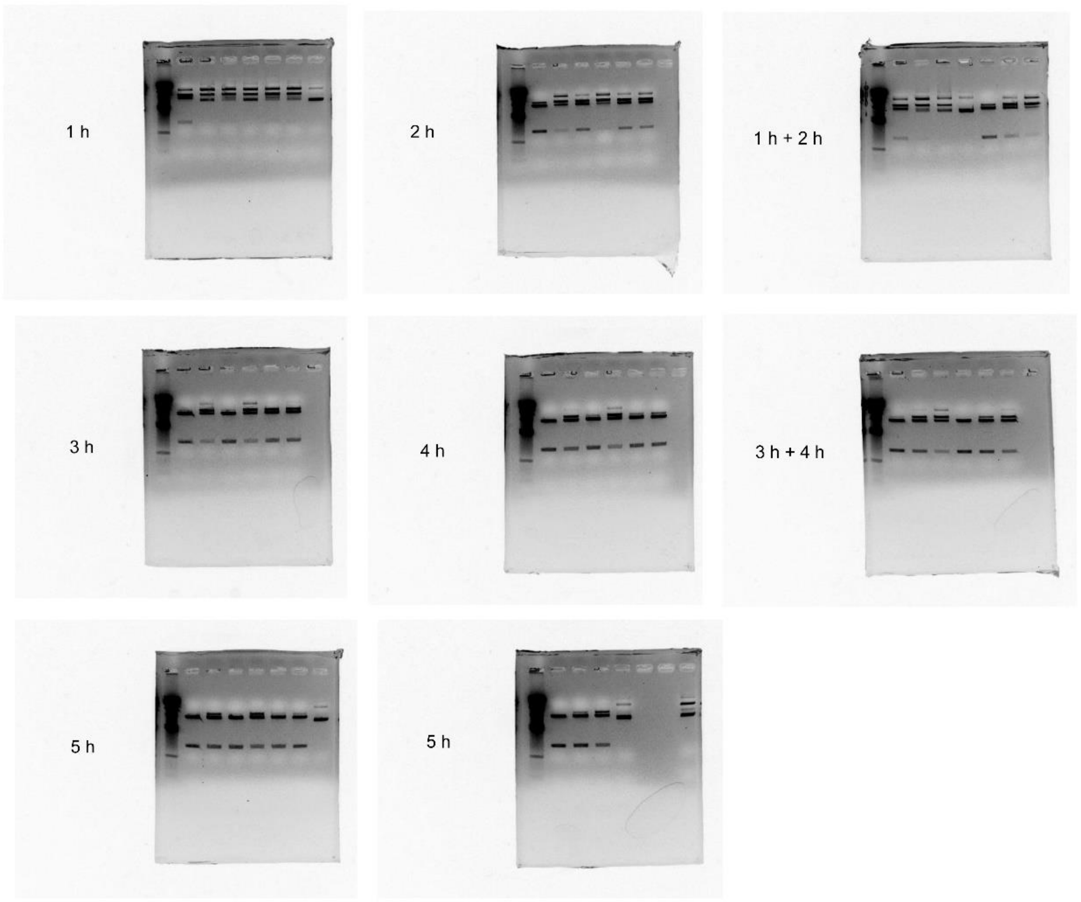

Supplementary Figure S1. Original gel images for Figure 3a.

Figure 5a

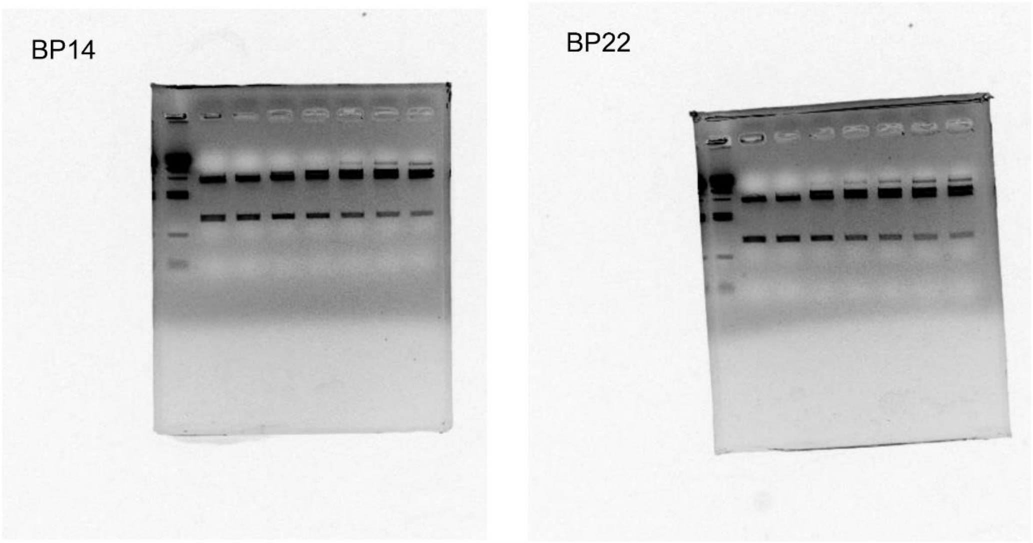

Supplementary Figure S2. Original gel images for Figure 5a.
